# Effect of Noncircular Channel on Distribution of Threshold Voltage in 3D NAND Flash Memory

**DOI:** 10.3390/mi14112007

**Published:** 2023-10-28

**Authors:** Donghyun Go, Gilsang Yoon, Jounghun Park, Donghwi Kim, Jiwon Kim, Jungsik Kim, Jeong-Soo Lee

**Affiliations:** 1 Department of Electrical Engineering, Pohang University of Science and Technology, Pohang 37673, Republic of Korea; godhyun@postech.ac.kr (D.G.); ygs6233@postech.ac.kr (G.Y.); mancity@postech.ac.kr (J.P.); kdh3879@postech.ac.kr (D.K.); jiwon104@postech.ac.kr (J.K.); 2 Department of Electrical Engineering, Gyeongsang National University, Jinju 52828, Republic of Korea

**Keywords:** 3D NAND flash memory, noncircular cell, spike, TCAD simulation, threshold voltage distribution, trapped charge

## Abstract

The instability in threshold voltage (*V_TH_*) and charge distributions in noncircular cells of three-dimensional (3D) NAND flash memory are investigated. Using TCAD simulation, we aim to identify the main factors influencing the *V_TH_* of noncircular cells. The key focus is on the nonuniform trapped electron density in the charge trapping layer (CTL) caused by the change in electric field between the circular region and the spike region. There are less-trapped electron (LT) regions within the CTL of programmed noncircular cells, which significantly enhances current flow. Remarkably, more than 50% of the total current flows through these LT regions when the spike size reaches 15 nm. We also performed a comprehensive analysis of the relationship between charge distribution and *V_TH_* in two-spike cells with different heights (H_Spike_) and angles between spikes (θ). The results of this study demonstrate the potential to improve the reliability of next-generation 3D NAND flash memory.

## 1. Introduction

Two-dimensional (2D) NAND flash memory, a conventional data storage technology, is characterized by the arrangement of memory cells in a two-dimensional structure for the storage and retrieval of data. Nonetheless, as the demand for increased storage capacity persists, and the relentless pursuit of cost reduction per bit continues, 2D NAND technology has encountered notable challenges that have impeded its progress. These limitations encompass scaling issues, restricted durability, and ongoing challenges in cost reduction during manufacturing. Consequently, the emergence of 3D NAND technology has become an imperative solution to surmount these challenges and extend the horizons delineated by Moore’s Law, primarily by achieving enhanced memory densities.

Three-dimensional (3D) NAND technology is primarily characterized by vertically stacked structures, forming a robust foundation for storing more data within a reduced physical footprint. A way to achieve improved capacity and cost efficiency in 3D NAND is to increase the word line (WL) stack [1,2,3,4]. The evolutionary changes in this architecture significantly transcend the limitations of traditional 2D NAND technology, effectively addressing the mounting demands for data storage and accessibility in modern computing devices.

Recently, 3D NAND technology has made remarkable progress with the adoption of the high-aspect-ratio (HAR) gate-all-around (GAA) structure, representing an advancement in NAND flash memory design. The GAA structure is designed to encompass the channel of a memory cell, thereby minimizing the gaps between memory cells and facilitating a higher cell packing density within the same physical space. This innovation translates to enhanced storage capacity within a given area, addressing the ever-increasing demand for more data storage. Moreover, the use of GAA polysilicon channels offers improved gate controllability and superior management of electrical fluctuations [5,6,7,8]. These advancements in control mechanisms have paved the way for the successful commercialization of triple-level cell (TLC) and quadruple-level cell (QLC) technologies within the domain of 3D NAND. This achievement has further enhanced the appeal and versatility of this memory technology [9,10,11,12]. However, it is essential to acknowledge that the transition to multilevel cells presents a unique set of challenges, particularly concerning threshold voltage (*V_TH_*) distribution. The narrower margins inherent in the programmed *V_TH_* distribution of multilevel cells introduce novel complexities and potential issues, diverging from the relative simplicity of single-level cells (SLCs) [13]. Incorporating the HAR structure into 3D NAND introduces a notable rise in process complexity, which, in turn, influences electrical performance and reliability [14,15,16,17]. This technology relies on the utilization of multiple stacked control gates and insulators crafted from alternating thin films of metal and silicon dioxide (SiO_2_). In this approach, a control gate is substituted for sacrificial silicon nitride (Si_3_N_4_), and holes within the stack are created through plasma etching. The procedure alternates between these thin layers of SiO_2_ and Si_3_N_4_. The cumulative stacking of these layers and the increasing number of successive layers result in a rapid rise in aspect ratio. Consequently, the fabrication of HAR structures presents its own set of challenges. The presence of small feature sizes and high plasma density during the manufacturing process pose potential risks. While it has been possible to achieve etching of up to 128 layers, this technique encounters limitations when pushed beyond this threshold, primarily due to the constraints of available plasma etching methods. The management of the HAR etch process is a complex undertaking, and it introduces a spectrum of profile distortions, including tapering, center-line tilt, and warping [18,19,20,21,22,23,24]. Simultaneously, a microtrench structure frequently forms close to the bottom region of the tapered etch hole. The cross-section of the microtrench generates a noncircular channel with spike-wise deformation, which can further degrade the uniformity of the electrical characteristics [25,26]. Although the electrical behavior of noncircular channel shape has been reported in some studies [27], it is not clear how the spike affects electrical properties of the cell and how *V_TH_* is determined by the spike.

Our study utilized Synopsys Sentaurus technology for computer-aided design (TCAD) to investigate the complexity of trap and channel inversion electron density (e-density) distributions within noncircular cells characterized by single or two spikes. Our objective is to establish a clear correlation between channel current density and *V_TH_* with respect to spike height and the angle between spikes. Furthermore, we intend to provide valuable insights into the *V_TH_* distribution of spiked noncircular cells and reveal how these structural complexities affect the electrical properties of memory cells.

## 2. Simulation Structure and Methods

**Figure 1:**
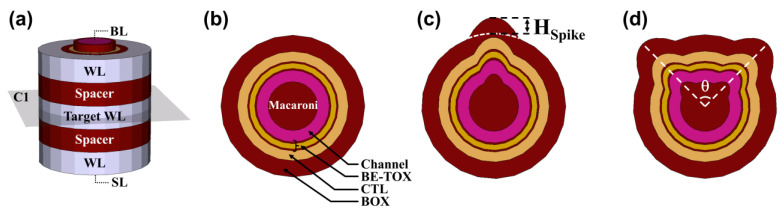
Schematic diagrams of simulated devices using gate-all-around (GAA) and vertical polysilicon (poly-Si) channel with macaroni channel structure. (**a**) Schematic diagrams of a 3D NAND flash memory string with three word lines (WLs), two spacers, a source line (SL), and a bit line (BL). Among the WLs, the middle WL is set as the target WL. (**b**) Cross-sectional schematics of the circular cell (C-cell), (**c**) single-spike cell (SSC), and (**d**) two-spike cell (TWSC).

Figure 1a presents a schematic diagram illustrating the structure of 3D NAND flash memory, featuring three word lines (WLs). Each WL is separated by the spacer, and the central WL is designated as the target WL. Both the WL length and spacer length are set to 30 nm. The radial structure of a cell is composed of a metal gate, blocking oxide (BOX), charge trap layer (CTL), and band-engineered tunneling layer (BE-TOX) consisting of O1/N1/O2, a polysilicon (poly-Si) channel, and macaroni oxide. At both ends of the poly-Si channel, there are the source line (SL) and bit line (BL). Additionally, Figure 1b–d show cross-sectional schematic diagrams of the circular cell (C-cell), single-spike cell (SSC), and two-spike cell (TWSC), respectively. To investigate the influence of the gap between spikes in TWSC cells, we introduced the parameter θ, as shown in Figure 1d. In this analysis, we varied the height of the spike (H_Spike_) and the angle between the two spikes (θ) over the ranges of 5 to 15 nm and 20° to 135°, respectively. Cell configurations featuring three or more spikes were not considered in this study due to the complexity associated with the etching process, which would require careful recalibration.

**Figure 2:**
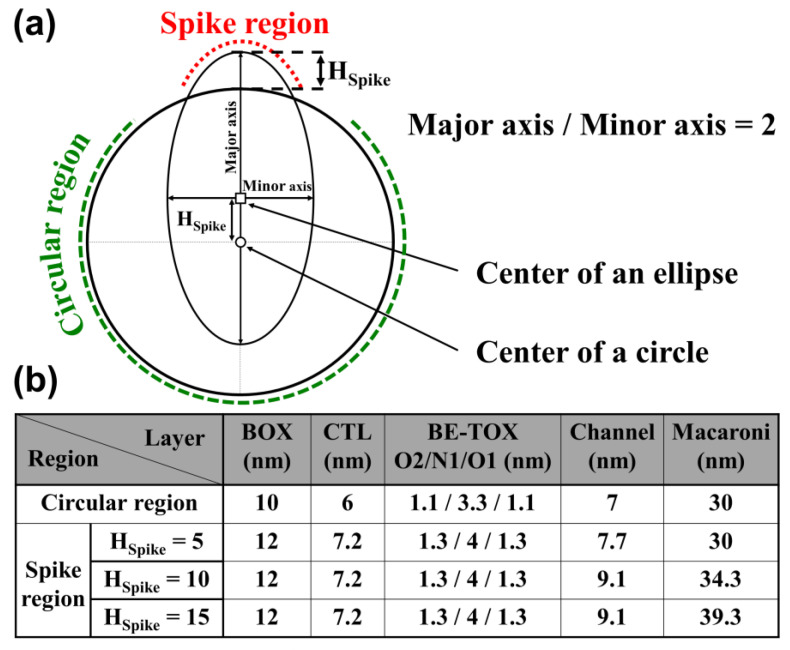
Description of noncircular cell: (**a**) Outline of a noncircular cell created by overlapping circle and ellipse. (**b**) Detailed values for the thickness of each layer (BOX, CTL, BE-TOX, channel, and macaroni) in circular and spike regions for H_spike_ = 5, 10, and 15 nm.

Figure 2 shows a detailed cross-sectional view of the spike cell used in the simulation. In our simulation, a portion of the ellipse’s structure is dedicated to shaping the spike shape. As shown in Figure 2a, the boundaries of the circular and elliptical shapes intersect, seamlessly creating the spike structure. Notably, the center of the ellipse is intentionally situated at a distance of H_Spike_ from the center of the circular shape. The outer perimeters of both the circle and the ellipse serve as the boundary between the metal gate and the BOX.

In accordance with the thickness of each layer, as shown in Figure 2b, the layers are assembled in the following order, from the outermost to the innermost: BOX, CTL, BE-TOX, channel, and macaroni. The thickness of each layer in the spike region was intentionally configured to be greater than that of the circular region. Consequently, the cell is separated into a spike region and a circular region.

The Hurkx Band-to-Band Tunneling model was employed for analyzing gate-induced drain leakage, along with the high field saturation mobility model applied to the channel region. To ensure accurate analysis of channel current, the quantum confinement effect was integrated into the study using the density-gradient model. In our assumptions, we considered that there were no defects present in the oxide layers within the BE-TOX and BOX layers. To replicate the program/erase (P/E) operation, transient simulations were carried out, employing a nonlocal tunneling model. This nonlocal tunneling model was applied to both electrons and holes at the interface between the channel and BE-TOX as well as at the junction of the metal gate and BOX during P/E operations. In the oxide layer, the effective tunneling mass for both electrons and holes was defined as 0.4. The carriers within the charge trap layer (CTL) were conveyed according to the drift-diffusion model, and the capture or emission of carriers into traps was described using the Shockley–Read–Hall (SRH) model. The material properties of the charge trap layer (CTL) employed in this study can be found in Table 1. The electron and hole traps in Si_3_N_4_ both exhibit gaussian energy distributions. The spatial distribution of the traps was maintained as constant since our primary focus was on evaluating alterations in electrical properties attributable to the noncircular channel shape.

For the program (PGM), erase (ERS), and read operations, the conditions were as follows: V_PGM_ = 16 V with t_PGM_ = 100 μs, V_ERS_ = −16 V with t_ERS_ = 1 ms, and V_PASS_ = 5 V, respectively. The voltage levels for the bit line (BL) and source line (SL) were maintained at 0.05 V and 0 V, respectively.

**Figure 3:**
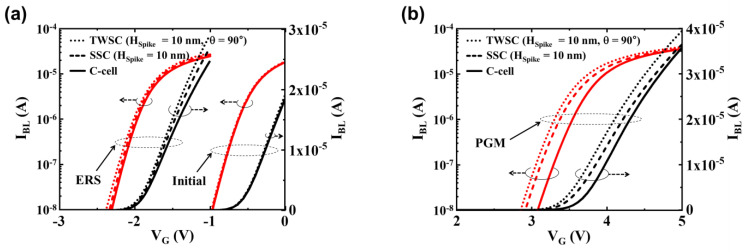
The bit-line current vs. gate voltage (I_BL_–V_G_) curves of the C-cell and noncircular cells plotted on the log and linear scales: (**a**) Initial, ERS, and (**b**) PGM states for the C-cell, SSC (H_Spike_ = 10 nm), and TWSC (H_Spike_ = 10 nm, θ = 90°) using 3D TCAD simulation.

These simulations were conducted in an environment with a temperature of 300 K, which is equivalent to room temperature. Figure 3 illustrates the bit-line current (I_BL_) plotted against the gate voltage (V_G_) for the C-cell and cells with spikes at various states, including the initial, ERS, and PGM states. These results were derived from the 3D TCAD simulations. The current curve is graphed with both logarithmic and linear scales. To extract the *V_TH_*, we employed the constant current method, identifying *V_TH_* as the voltage at which 2 μA flows through the bit line (BL). In the initial, ERS, and PGM states, the *V_TH_* values for the C-cell were measured at −0.5 V, −2 V, and 3.67 V, respectively. To explore the variation in *V_TH_* as influenced by the number of spikes, a comparison was made between the C-cell, SSC (with H_Spike_ = 10 nm), and TWSC (with H_Spike_ = 10 nm and θ = 90°). In the case of the SSC and TWSC cells, it was observed that the *V_TH_* values in the initial and ERS states exhibited minimal differences when compared to the *V_TH_* of the C-cell, as depicted in Figure 3a. Nevertheless, during the PGM state, a noticeable negative shift in *V_TH_* was distinctly observed for noncircular cells due to structural deformations, which is evident in Figure 3b. It is worth noting that TWSC cells with θ = 90° exhibited a lower *V_TH_* compared to SSC. To gain a deeper understanding of their electrical behaviors, SSC and TWSC cells were subjected to simulations with varying H_Spike_ and θ values.

## 3. Results and Discussion

### 3.1. SSC Characteristics

**Figure 4:**
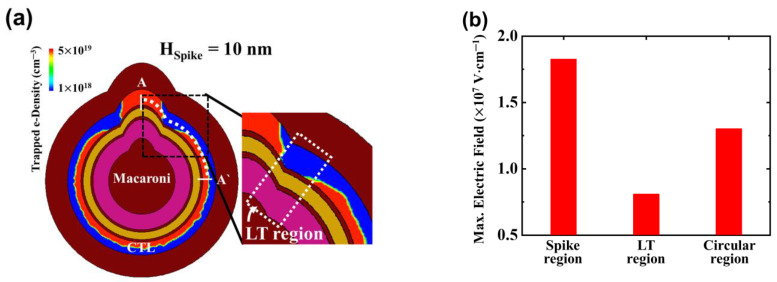
(**a**) Cross-sectional distribution of the trapped electron density (e-density) in the CTL in the PGM state of the SSC with 10 nm of H_spike_ and (**b**) maximum vertical electric field (*E_max_*) extracted in the O1 layer of the BE-TOX layer by different regions (spike, LT, circular) during the PGM operation.

In Figure 4a, the distribution of trapped e-density within the charge trap layer (CTL) during the PGM state of the SSC is depicted. Notably, the trapped e-density in the CTL within the spike region surpasses that within the circular region. There is an observable gradual decline in trapped e-density within the space situated between the spike and circular regions. This region, where the trapped e-density experiences a reduction of 10% compared to the circular region, is now defined as the less-trapped electron (LT) region. It is essential to highlight that in the SSC, two distinct LT regions are formed.

In Figure 4b, the diagram illustrates the maximum electric field (*E_max_*) on the O1 layer of BE-TOX at V_PGM_ = 16 V in the SSC with H_Spike_ = 10 nm. In accordance with Gauss’s law, the relationship between the electric field of BE-TOX and BOX, denoted as *E_BE-TOX_* and *E_BOX_* respectively, is expressed as *E_BE-TOX_* = *E_BOX_* (*R + EOT*)/*R*, where *R* is the radius of curvature [28]. Given that the spike region exhibits a smaller R in comparison to the circular region, *E_BE-TOX_* in the spike region is notably higher. *E_BE-TOX_* directly impacts electron tunneling probability and trapped e-density during the PGM operation.

The concave shape observed in the LT region has the potential to reduce *E_max_* and induce a lower quantity of trapped charges within the LT region.

**Figure 5:**
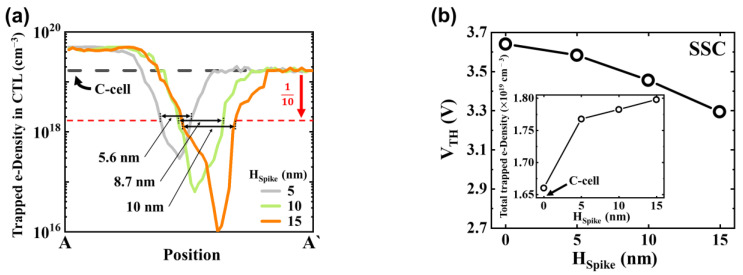
(**a**) Concentration of the trapped electron density (e-density) in CTL with different H_Spike_ values (5, 10, and 15 nm) along the A-A′ perimeter. The less-trapped electron (LT) region, where the e-density decreases by 10% of the circular region, is indicated with the red dashed line. (**b**) *V_TH_* of the C-cell (H_Spike_ = 0 nm) and the SSC in the PGM state with different H_Spike_ values (0, 5, 10, and 15 nm). Inset: total trapped e-density vs. H_Spike_ value.

In Figure 5a, the diagram shows the distribution of the average trapped e-density during the PGM state along the A-A′ perimeter, taking into account varying H_Spike_ values. The average trapped e-density was determined by radially integrating the cumulative charge at each position within the charge trap layer (CTL). In the case of the circular cell (C-cell), a consistent and uniform trapped e-density of 1.6 × 10^19^ cm^−3^ was established. As a result, the LT region in spike cells is defined as an area with a trapped e-density lower than 1.6 × 10^18^ cm^−3^. For the SSC, a fully trapped e-density of 5 × 10^19^ cm^−3^ was identified in the spike region, while the circular region of SSC shared the same trapped e-density value as the C-cell. With an increase in H_Spike_, the minimum trapped e-density decreased, and the LT region expanded from 5.6 to 10 nm. The distribution of trapped e-density is inherently dependent on the H_Spike_ values, which in turn affect the current flow within the channel and the corresponding *V_TH_* values. As illustrated in Figure 5b, SSC exhibits a lower *V_TH_* compared to the C-cell, and this value further decreases as H_Spike_ increases. In general, C-cells with lower trapped e-density within the CTL tend to have smaller *V_TH_* values. However, as the H_Spike_ of SSC increases, the overall trapped e-density within the CTL increases, thus contradicting the trend observed for *V_TH_* in C-cells.

To elucidate the reason behind this contrasting *V_TH_* trend in noncircular cells, a more comprehensive examination of the connection between trapped e-density in spike cells and channel current density, which directly influences *V_TH_*, is imperative.

**Figure 6:**
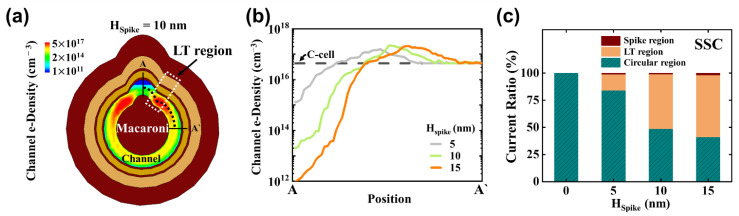
(**a**) Cross-sectional distribution of the channel e-density with H_Spike_ = 10 nm at *V_read_* = *V_TH_*; (**b**) channel e-density with different H_Spike_ along A-A′ perimeter; (**c**) ratio of current flowing through circular, LT, and spike regions to total channel current (I_BL_ = 2 μA) with different H_Spike_ values (0, 5, 10, and 15 nm).

Figure 6a provides a cross-sectional view of channel electron distribution within the SSC with H_Spike_ = 10 nm, observed at *V_read_* = *V_TH_*. Despite the uniform application of *V_read_* across all regions, the spike regions with high trapped e-density result in locally smaller channel e-density in comparison to other channel regions. However, the LT region, characterized by lower trapped e-density in the CTL, exhibits an increased channel electron count and subsequently a higher local current density. Figure 6b presents the average channel e-density along the A-A′ perimeter, considering various H_Spike_ values. The channel e-density for the C-cell is calculated to be 4.6 × 10^16^ cm^−3^. Notably, the spike region displays the lowest local current, and this low current further decreases as H_Spike_ values increase. The channel e-density within the circular region aligns with that of the C-cell. A local e-density peak is evident in the LT region, and this peak coincides with the position of the minimum trapped e-density in the CTL (as shown in Figure 5a). With the increase in H_Spike_, the local e-density peak value rises from 1.6 × 10^17^ to 2.1 × 10^17^ cm^−3^. Figure 6c presents the proportion of currents flowing within the three regions concerning the total channel current (at *V_read_* = *V_TH_*) in programmed SSCs. The total channel current amounts to 2 μA. Remarkably, less than 1% of the current is directed through the spike region. In the case of a circular cell (C-cell) with H_Spike_ = 0 nm, the total current belongs to the circular region. However, as H_Spike_ values increase, there is a notable rise in local current within the LT region. The normalized perimeter length, determined at H_Spike_ = 15 nm, indicates that 70% of the current is attributed to the circular region, 18% to the spike region, and 12% to the LT region. Despite the LT region having the smallest spatial area among the three regions, in SSCs with H_Spike_ = 15 nm, approximately 57% of the total current flows through the LT region.

In conclusion, it can be inferred that a relatively high current is directed towards the LT region in the programmed spike cell, ultimately resulting in a low VTH for the spike cell.

### 3.2. TWSC Characteristics

**Figure 7:**
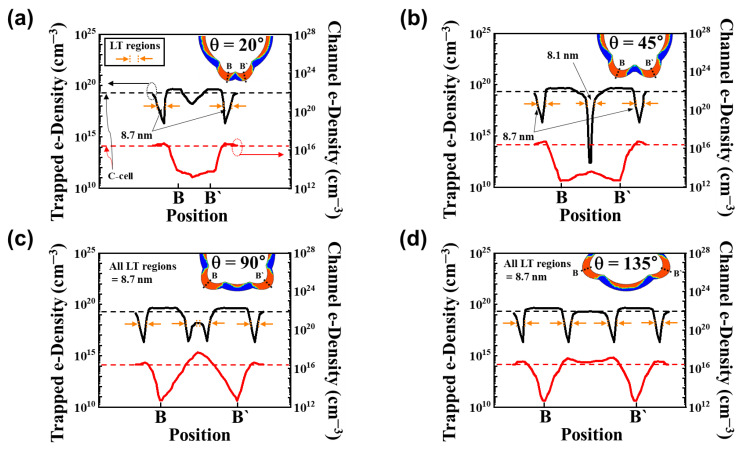
TWSC with H_Spike_ = 10 nm and θ for (**a**) 20°, (**b**) 45°, (**c**) 90°, and (**d**) 135° in PGM state. The trapped e-density in the CTL and channel e-density with different θ values along the B-B′ perimeter are shown. Each inset indicates the corresponding cross-sectional distribution of the trapped e-density in the CTL of programmed TWSCs.

Figure 7 provides an overview of the trapped e-density within the CTL and the channel e-density at *V_read_* = *V_TH_* in the vicinity of two spikes within the programmed TWSCs. In this simulation, H_Spike_ is kept constant at 10 nm, while the parameter θ is adjusted within the range of 20° to 135°. The insets display the cross-sectional distribution of trapped electrons in the CTL. Observations reveal that at θ = 135°, four distinct LT regions are identified, with two of these LT regions positioned within the B-B′ region (as shown in Figure 7d). Each LT region spans a length of 8.7 nm. Within all four LT regions, the location of minimum trapped e-density coincides with the peak location of local channel e-density. The two LT regions positioned between the spikes exhibit a minimum trapped e-density of 2.1 × 10^16^ cm^−3^, and the peak channel e-density is 1.3 × 10^17^ cm^−3^. As θ decreases to 90°, the two LT regions positioned between the spikes draw closer together, with no alteration in their length. As the circular region between the LT regions narrows, the relative proportion of LT regions within the B-B′ region increases significantly. Consequently, while the minimum trapped e-density remains unchanged, the peak channel e-density increases to 5 × 10^17^ cm^−3^ (as shown in Figure 7c). Further reducing the angle to θ = 45° in the TWSCs results in the merging of the two LT regions positioned between the spikes. At θ = 45°, there is a single LT region with a length of 8.1 nm between the spikes. The region situated between the two spikes exhibits a structure characterized by a low electric field, resulting in reduced levels of both trapped e-density in the CTL and channel e-density (as shown in Figure 7b). Upon reaching θ = 20°, the analysis reveals the presence of only two LT regions, each with a length of 8.7 nm. At both θ = 45° and θ = 20° within the TWSC, there is a notable absence of e-density between the two spikes in comparison to the circular region. This observation suggests that the majority of the channel current flows through the circular region and the two LT regions (as shown in Figure 7a). Furthermore, the spike region at θ = 20° is narrower in comparison to θ = 45°, indicating that the area experiencing current flow is broader.

**Figure 8:**
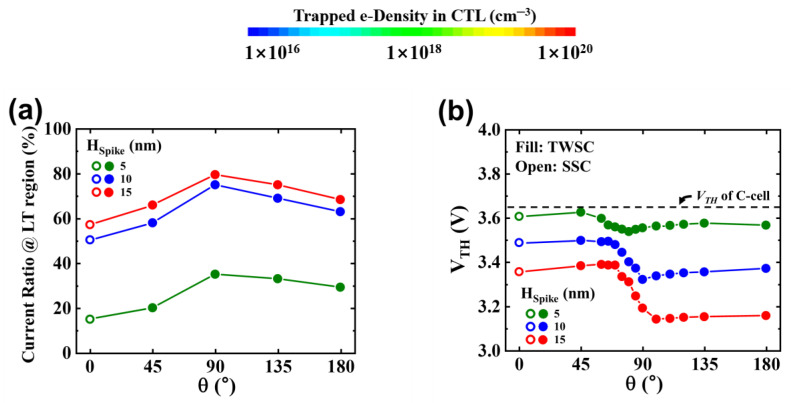
The electrical characteristics (current ratio and threshold voltage) as functions of H_Spike_ and θ for the TWSCs. (**a**) Ratio of current flowing in the LT regions to total channel current (I_BL_ = 2 μA) with the H_Spike_ and θ of TWSCs and (**b**) threshold voltage (*V_TH_*) in programmed TWSCs depending on H_Spike_ and θ of spikes.

Figure 8a illustrates the current ratio that flows through the LT regions relative to the total current as a function of θ. Notably, the current in the spike region remains less than 1%, emphasizing that the majority of the channel current is directed through the LT and circular regions. In the TWSC, the current ratio reaches its maximum at θ = 90°, irrespective of the spike’s size. This ratio increases from 40% for H_Spike_ = 5 nm to 80% for H_Spike_ = 15 nm. At θ = 180°, where four LT regions are present, the current ratio is higher compared to θ = 0°, which has only two LT regions. Figure 8b presents the *V_TH_* distribution for TWSCs with different H_Spike_ and θ values. The dashed line represents the *V_TH_* value of the C-cell. All SSCs and TWSCs exhibit lower *V_TH_* values than the C-cell, with a larger spike inducing a more substantial decrease in *V_TH_*. Particularly, around θ = 90° in the TWSC cell, the noticeable increase in channel e-density between the spikes contributes to the reduced *V_TH_* (as shown in Figure 7c). As the size of the spike increases, the *V_TH_* distribution expands within the noncircular cell, leading to increased *V_TH_* instability. Mitigation of the significant *V_TH_* instability caused by the spike can be achieved through the implementation of a rigorous Incremental Step Pulse Program (ISPP) method. However, it is essential to strike a balance between *V_TH_* distribution and PGM time when optimizing the ISPP conditions, as longer PGM times may lead to reduced operational speed. To enhance *V_TH_* consistency, reducing the curvature in the spike region to achieve a uniform E-field is recommended. It is important to adjust the curvature when the layer thickness in the spike region changes. Controlling layer thickness to minimize *V_TH_* variability in spike cells is an area of focus for future research and development.

## 4. Conclusions

This study has delved into the instability and charge distribution of the threshold voltage (*V_TH_*) in noncircular cells of three-dimensional (3D) NAND flash memory. Utilizing TCAD simulations with spike cells, we effectively pinpointed the crucial factors that exert significant influence on *V_TH_*. A major focus is placed on the role of electric field fluctuations, which give rise to non-uniform trapped e-density within the charge trapping layer (CTL). Our investigation revealed the presence of regions with less-trapped (LT) regions within the CTL of programmed noncircular cells, resulting in increased current flow. Furthermore, we conducted a comprehensive analysis of the relationship between charge distribution and *V_TH_* for two-spike cells with varying heights of spike (H_Spike_) and angle between the spikes (θ). These findings clearly demonstrate that *V_TH_* instability in noncircular cells intensifies as H_Spike_ values increase. The results of this study offer a comprehensive understanding of *V_TH_* instability in noncircular cells, provide valuable insights for optimizing 3D NAND flash memory techniques, and furnish guidelines for enhancing both reliability and performance.

## Figures and Tables

**Table 1:** Material properties of silicon nitride used in CTL.

Parameter	Value
Bandgap	5.0 eV
Peak Energy Level of Electron Trap	1.0 eV
Standard Deviation of Electron Trap	0.1 eV
Total Density of Electron Trap ($N_{T}$)	${5\times 10^{19}\;cm}^{-3}$
Peak Energy Level of Hole Trap	2.5 eV
Standard Deviation of Hole Trap	0.1 eV
Total Density of Hole Trap	${5\times 10^{18}\;cm}^{-3}$
